# Psychological Distress, Stress, and Personality Traits in Patients Undergoing Chronic Hemodialysis: A Comparative Psychometric Study

**DOI:** 10.3390/bs16030423

**Published:** 2026-03-14

**Authors:** Simona Nicoleta Neagu, Aniella Mihaela Vieriu

**Affiliations:** The Department of Teacher Training and Social Sciences, The National University of Science and Technology POLITEHNICA, 060042 Bucharest, Romania; simona.neagu@upb.ro

**Keywords:** hemodialysis, renal insufficiency, chronic, depression, anxiety, psychological distress, personality traits, stress, psychological

## Abstract

Psychological comorbidity is increasingly recognized as a critical factor influencing outcomes in chronic illness management, particularly in patients with end-stage renal disease (ESRD). The present study examines the psychological burden associated with long-term hemodialysis in patients with ESRD, focusing on emotional distress and maladaptive personality traits. Specifically, it explores group differences between hemodialysis patients and matched healthy controls in levels of stress, anxiety, depression, and psychopathological tendencies, including neuroticism, paranoia, and psychopathy-related traits, as well as exploratory associations with treatment duration. A purposive sample of 60 participants (30 patients undergoing chronic hemodialysis and 30 age- and sex-matched healthy controls) was assessed using validated psychometric instruments: The Hospital Anxiety and Depression Scale, the Pichot Neuroticism and Psychopathy Questionnaire, and a 23-item stress measurement questionnaire adapted to the dialysis context. Both descriptive and inferential statistical analyses were conducted, including independent-samples *t*-tests and effect size calculations (Cohen’s *d*). Compared to healthy controls, hemodialysis patients exhibited significantly higher levels of psychological distress across multiple domains. Large between-group effect sizes were observed for depression (Cohen’s *d* = 1.26) and perceived stress (*d* = 1.51), while moderate effects were identified for anxiety (*d* = 0.70), neuroticism (*d* = 0.58), and psychopathy-related traits (*d* = 0.82). Exploratory analyses indicated that patients with less than 10 years of dialysis experience reported significantly higher stress levels than those with longer treatment duration, whereas differences in anxiety, depression, and personality traits by dialysis duration were not statistically significant. These findings highlight the substantial emotional burden associated with long-term hemodialysis and underscore the importance of routine psychological screening and early psychosocial interventions to support adaptation, treatment adherence, and quality of life in nephrology care.

## 1. Introduction

End-stage renal disease (ESRD) represents a growing global health concern, with prevalence rates continuing to rise and imposing substantial physical, psychological, and socioeconomic burdens on affected individuals (Abbas et al., 2024; Doan et al., 2025). Hemodialysis remains an essential long-term treatment; however, its demanding schedule, strict lifestyle constraints, and ongoing dependence on medical technology profoundly shape patients’ daily routines, sense of autonomy, and emotional well-being (Lu et al., 2024; Zhang et al., 2024). Physical burdens such as fatigue and chronic pain exacerbate psychological strain (Bossola et al., 2011; Lopes et al., 2007; Weisbord et al., 2005). The cumulative effects of these constraints often manifest as persistent stress, anxiety, depressive symptoms, and reduced health-related quality of life (HRQoL) (Feroze et al., 2012; Song & Zhang, 2024; Yu et al., 2021).

A growing body of research indicates that psychological well-being plays a critical role in treatment adaptation, self-management behaviors, and overall outcomes in ESRD. Nevertheless, emotional distress and broader psychological vulnerabilities remain insufficiently addressed in routine nephrology care (Huang et al., 2020). Depressive symptoms are reported in approximately 30–50% of patients undergoing hemodialysis, while clinically relevant anxiety affects an estimated 20–30% (Bagasha et al., 2021; Chang & Kim, 2025). Beyond their impact on subjective well-being, these emotional difficulties are associated with poorer treatment adherence, increased healthcare utilization, and diminished quality of life (Yu et al., 2021; Zhang et al., 2024).

Importantly, psychological functioning in ESRD is not limited to transient emotional states. Stable individual differences—such as personality traits related to emotional reactivity, interpersonal sensitivity, or behavioral regulation—may shape how patients perceive, interpret, and cope with the chronic demands of dialysis (Chilcot et al., 2010; Harenski et al., 2009). These tendencies reflect subclinical elevations along normative personality dimensions rather than clinical psychopathology. From a behavioral science perspective, subclinical variations may modulate stress responses, influence coping strategies, and contribute to differential patterns of adjustment over time.

Recent international studies emphasize that the interaction between personality structure and chronic illness-related stressors plays a meaningful role in psychological adaptation among patients with ESRD (Bazrafshan et al., 2023; Touil et al., 2023; Uzdil et al., 2023). Higher levels of neuroticism, lower conscientiousness, and reliance on maladaptive coping strategies have been linked to increased emotional distress and poorer HRQoL, whereas resilience, optimism, and problem-focused coping are associated with more favorable psychological adjustment (Cukor et al., 2007; Kovacs et al., 2011). The results suggest the relevance of incorporating trait-level psychological assessment alongside traditional symptom-focused screening in dialysis care, allowing for early identification of patients who may be more vulnerable to stress exposure.

Validated psychometric instruments provide valuable tools for capturing both current emotional distress and enduring personality-related vulnerabilities. The Hospital Anxiety and Depression Scale (HADS) is widely used to assess anxiety and depressive symptoms in medical populations, while the Pichot Neuroticism and Psychopathy Questionnaire offers insight into normative personality tendencies related to emotional instability, suspiciousness, and behavioral regulation (Anastasi & Urbina, 1997; Pichot, 1987; Zigmond & Snaith, 1983). Elevated scores indicate relative predispositions along a continuum, not clinical diagnoses. Their combined use allows for a comprehensive behavioral and psychological profile of individuals undergoing long-term hemodialysis, supporting more personalized psychosocial interventions.

### 1.1. Theoretical Background and Rationale

International literature consistently documents elevated psychological distress among patients with ESRD, with recent findings indicating heightened vulnerability during periods of external stress, such as the COVID-19 pandemic (Cordoș et al., 2024; Yu et al., 2021). However, much of the existing research focuses narrowly on anxiety and depression, with limited attention to broader subclinical personality-related tendencies—such as heightened suspiciousness or emotional dysregulation—that may further influence coping and adjustment to chronic treatment demands. In addition, cross-cultural variations in healthcare systems, social support, and coping norms may affect psychological responses to dialysis, limiting the generalizability of findings across populations (Soponaru et al., 2016).

In Romania, empirical research addressing the psychological dimensions of chronic hemodialysis remains relatively scarce. Available studies suggest elevated stress levels, reduced HRQoL, and a predominance of emotion-focused coping strategies among dialysis patients; however, few investigations have systematically examined subclinical personality correlates or integrated multiple validated psychometric measures within a single framework (Cordoș et al., 2024; Soponaru et al., 2016). This gap constrains the development of culturally informed psychosocial interventions tailored to the needs of Romanian ESRD populations.

The present study seeks to address these limitations by adopting a multidimensional behavioral science approach that integrates measures of perceived stress, emotional symptoms, and subclinical personality-related tendencies. By comparing patients undergoing chronic hemodialysis with healthy controls, the study aims to clarify the associations between subclinical personality tendencies and emotional distress, including how these traits interact with dialysis-specific stressors. Dialysis duration is examined in an exploratory manner to assess whether prolonged exposure to treatment demands may be associated with differences in perceived stress, while acknowledging the limitations imposed by subgroup size.

### 1.2. Study Aim and Hypotheses

The primary aim of this study is to explore psychological and psychopathological correlates of chronic hemodialysis in patients with ESRD, with a focus on perceived stress, anxiety, depression, and maladaptive personality-related tendencies—specifically neuroticism, paranoid ideation, and psychopathy-related traits—compared to a healthy control group.

General hypothesis: Prolonged exposure to the psychosocial demands associated with ESRD and chronic hemodialysis is associated with higher levels of emotional distress and a greater expression of maladaptive personality-related tendencies relative to healthy individuals.

Specific hypotheses:

**H1.**  *Patients undergoing hemodialysis report significantly higher perceived stress levels than healthy controls.*

**H2.**  *Anxiety and depressive symptoms are more prevalent among hemodialysis patients.*

**H3.**  *Personality-related tendencies associated with neuroticism, paranoid ideation, and psychopathy-related traits are more pronounced in the patient group.*

**H4** **(Exploratory).** *Differences in perceived stress may emerge between patients with shorter (<10 years) and longer (≥10 years) durations of hemodialysis, though conclusions are considered hypothesis-generating due to sample size limitations.*

By integrating emotional, cognitive, and personality-related dimensions, this study contributes to a more nuanced behavioral understanding of psychological adaptation in ESRD and supports the implementation of systematic psychological screening and psychosocial support within nephrology care.

## 2. Materials and Methods

### 2.1. Study Design

The present study employed a cross-sectional, case–control design to examine psychological distress and personality-related tendencies in patients undergoing chronic hemodialysis compared to a non-clinical control group.

The independent variable was group membership (hemodialysis patients vs. healthy controls).

The dependent variables included perceived stress, anxiety, depressive symptoms, and personality-related tendencies (neuroticism, paranoid ideation, and psychopathy-related traits).

Dialysis duration (<10 years vs. ≥10 years) was examined as an exploratory grouping variable within the clinical sample.

### 2.2. Participants and Sampling

A non-probabilistic purposive sampling strategy was used to recruit participants who met the study’s inclusion criteria. The final sample consisted of 60 adults, divided into two groups:

Hemodialysis group (n = 30): Patients diagnosed with end-stage renal disease and undergoing chronic hemodialysis, recruited from the Hemodialysis Department of Dr. Carol Davila Clinical Hospital, Bucharest, Romania.

Control group (n = 30): Community-dwelling adults recruited from the general population, including employees of The National University of Science and Technology POLITEHNICA Bucharest and Metrorex S.A.

The control group was selected to approximate the clinical group in terms of age and educational level, while excluding individuals with known chronic medical or psychiatric conditions.

Inclusion criteria: Age between 18 and 65 years; Ability to understand and complete self-report questionnaires; For the clinical group: ongoing hemodialysis treatment; For controls: absence of diagnosed chronic medical or psychiatric illness.

Exclusion criteria: Documented severe psychiatric disorders (e.g., psychotic disorders); Cognitive impairment preventing questionnaire completion.

Within the hemodialysis group, participants were further categorized based on treatment duration (<10 years vs. ≥10 years) for exploratory analyses.

### 2.3. Ethical Considerations

This study was conducted in accordance with the Declaration of Helsinki (World Medical Association, 1975/2013) and was approved by the Scientific Research Ethics Committee of the National University of Science and Technology POLITEHNICA Bucharest (Approval No. 92; Approval Date: 31 January 2025).

Participants undergoing hemodialysis were recruited with the support of the clinical psychologist working within the dialysis center, who informed eligible patients about the study and provided the questionnaire link only to those who voluntarily expressed interest in participating. The research team had no direct contact with potential participants prior to their consent.

The questionnaire was administered online via Google Forms. Before accessing the questionnaire, participants were presented with detailed study information, including the purpose of the research, voluntary nature of participation, right to withdraw at any time, and assurances of anonymity and confidentiality.

Due to the anonymous and minimal-risk design of the study, written informed consent was not required. Informed consent was obtained electronically through the participants’ voluntary completion and submission of the questionnaire, in accordance with the approval of the Ethics Committee.

### 2.4. Research Instruments

The Hospital Anxiety and Depression Scale (HADS; Zigmond & Snaith, 1983) is a 14-item self-report questionnaire designed to assess anxiety and depressive symptoms in medical populations while minimizing confounding from somatic symptoms. It consists of two subscales (Anxiety and Depression), each containing seven items rated on a 4-point Likert scale. The scale was originally developed by Zigmond and Snaith and published by the NFER-Nelson Publishing Company (Windsor, UK). The instrument demonstrates good reliability and validity across clinical populations.

The Pichot Neuroticism and Psychopathy Questionnaire (PNP; Pichot, 1987) assesses personality-related tendencies across three dimensions: neuroticism, paranoid ideation, and psychopathy-related traits. The instrument was originally developed by Pierre Pichot at Hôpital Sainte-Anne (Paris, France). In the present study, the PNP was used to capture personality-related tendencies within a psychometric framework rather than clinical diagnoses of personality disorders.

Dialysis-Specific Stress Questionnaire. The dialysis-specific stress questionnaire was developed for research purposes to assess perceived stress related to health status, daily functioning, and treatment-related burden. Item content was generated based on clinical experience, review of relevant literature on dialysis-related stress, and consultation with healthcare professionals working in nephrology settings. The questionnaire was administered to both hemodialysis patients and healthy controls to allow comparative analysis of perceived stress levels. Items are rated on a 5-point Likert scale. Preliminary psychometric validation was conducted in a pilot sample of 30 hemodialysis patients following classical test theory principles, including item difficulty, item discrimination, item-total correlations, and internal consistency analysis. Although the instrument demonstrated excellent internal consistency in the present sample (Cronbach’s α = 0.94), further research is needed to establish its construct validity and factor structure. The full instruments are provided in Appendix A. The Dialysis-Specific Stress Questionnaire is a study-specific instrument; details regarding its psychometric validation are provided in Appendix B.

### 2.5. Analysis

Data were analyzed using descriptive and inferential statistics to compare psychological outcomes between patients undergoing chronic hemodialysis and healthy controls. Statistical analyses were conducted using IBM SPSS Statistics for Windows, Version 22 (IBM Corp., Armonk, NY, USA). Descriptive statistics (means and standard deviations) summarized anxiety, depression, stress, and personality-related variables. Group differences were examined using independent samples *t*-tests, including exploratory comparisons based on dialysis duration (<10 years vs. ≥10 years). Assumptions of normality and homogeneity of variance were assessed prior to analysis. Statistical results are reported with corresponding effect sizes (Cohen’s *d*). In addition, 95% confidence intervals for effect sizes were calculated to provide an estimate of the magnitude and precision of group differences. Given the exploratory nature of the study and the modest sample size, no formal correction for multiple comparisons was applied; therefore, results should be interpreted with appropriate caution.

## 3. Results

### 3.1. Sample Characteristics

The final sample consisted of 60 participants, divided equally into a hemodialysis group (n = 30) and a healthy control group (n = 30). In the hemodialysis group, 19 participants were males and 11 females, while the control group included 17 males and 13 females. The gender distribution was relatively balanced across groups.

Regarding educational level, the groups were broadly comparable. Among patients undergoing hemodialysis, 14 participants (46.7%) had completed higher education, 15 (50%) had completed high school, and one participant (3.3%) had completed secondary education only. In the control group, 15 participants (50%) had completed higher education, and 15 (50%) had completed high school.

Within the hemodialysis group, most participants reported no prior history of psychological difficulties before the onset of chronic kidney disease (93.3%). A subset of patients (36.7%) reported previous medical conditions commonly associated with chronic illness, such as diabetes, hypertension, or cardiovascular disease. These data were collected to contextualize psychological vulnerability and to distinguish between pre-existing conditions and those potentially emerging during long-term treatment.

The duration of hemodialysis treatment varied widely, ranging from less than one year to more than 20 years, reflecting substantial heterogeneity in treatment experience. For exploratory analyses, patients were grouped according to dialysis duration into those receiving treatment for less than 10 years (n = 15) and those receiving treatment for 10 years or longer (n = 15).

### 3.2. Anxiety and Depression (HADS)

Anxiety and depression were assessed using the Hospital Anxiety and Depression Scale (HADS) in patients undergoing chronic hemodialysis (n = 30) and matched healthy controls (n = 30).

Hemodialysis patients reported significantly higher anxiety scores (M = 10.93, SD = 4.77) compared to healthy controls (M = 8.20, SD = 2.81), t(58) = 2.86, *p* = 0.006, Cohen’s *d* = 0.70, 95% CI [0.18, 1.22], indicating a moderate effect size (Table 1).

Similarly, depression scores were significantly higher in the hemodialysis group (M = 10.90, SD = 5.01) than in controls (M = 5.67, SD = 3.04), t(58) = 4.57, *p* < 0.001, Cohen’s *d* = 1.26, 95% CI [0.71, 1.81], indicating a large effect size.

Exploratory analyses examining dialysis duration (<10 years vs. ≥10 years) revealed no statistically significant differences in anxiety (*p* = 0.230) or depression (*p* = 0.478).

### 3.3. Personality Traits (PNP)

Personality traits were assessed using the Pichot Neuroticism and Psychopathy Questionnaire.

Hemodialysis patients demonstrated significantly higher neuroticism scores (M = 65.77, SD = 11.45) compared to controls (M = 59.13, SD = 11.37), t(58) = 2.29, *p* = 0.025, Cohen’s *d* = 0.58, 95% CI [0.06, 1.10], indicating a moderate effect size (Table 2).

Psychopathy-related trait scores were also significantly higher in hemodialysis patients (M = 62.57, SD = 8.73) compared to controls (M = 53.97, SD = 12.04), t(58) = 2.76, *p* = 0.008, Cohen’s *d* = 0.82, 95% CI [0.29, 1.35], indicating a large effect size.

Paranoid trait scores were higher in the hemodialysis group (M = 56.40, SD = 18.12) compared to controls (M = 51.30, SD = 11.55), but this difference did not reach statistical significance, t(58) = 1.29, *p* = 0.202, with a small effect size (Cohen’s *d* = 0.34) and a 95% confidence interval [−0.17, 0.85], suggesting a modest and imprecisely estimated difference.

For descriptive purposes, elevated neuroticism scores (T > 70) were observed in 57% (n = 17) of hemodialysis patients compared to 23% (n = 7) of controls. Elevated psychopathy-related scores were present in 37% (n = 11) of hemodialysis patients and 13% (n = 4) of controls. Elevated paranoid trait scores were observed in 40% (n = 12) of hemodialysis patients compared to 17% (n = 5) of controls. These categorizations reflect elevated trait levels based on psychometric conventions and do not represent clinical diagnoses. Reporting both continuous scores and categorical distributions is intended to enhance transparency, particularly given the modest sample size.

Exploratory subgroup analyses based on dialysis duration revealed no statistically significant differences for neuroticism (*p* = 0.734), paranoid tendencies (*p* = 0.481), or psychopathic traits (*p* = 0.889).

### 3.4. Dialysis-Specific Stress

Dialysis-specific stress was assessed in both hemodialysis patients and healthy controls to allow comparative analysis. Hemodialysis patients reported significantly higher stress scores (M = 62.40, SD = 20.83) compared to healthy controls (M = 37.23, SD = 11.14), t(58) = 5.84, *p* < 0.001, Cohen’s *d* = 1.51, 95% CI [0.93, 2.09], indicating a large effect size (Table 3).

Within the hemodialysis group, patients receiving treatment for less than 10 years reported significantly higher stress levels (M = 71.27, SD = 20.58) than those undergoing dialysis for 10 years or more (M = 53.53, SD = 17.50), t(28) = −2.54, *p* = 0.017.

Given the study-specific nature of the instrument and its preliminary psychometric validation, these findings should be interpreted with appropriate caution.

## 4. Discussion

This study examined psychological outcomes—including dialysis-specific stress, affective symptoms (anxiety and depression), and personality traits—in patients undergoing chronic hemodialysis compared to healthy controls. Four hypotheses guided this research, focusing on stress prevalence, affective symptoms, personality traits, and the influence of dialysis duration on psychological outcomes.

### 4.1. Hypotheses Overview

H1—Stress prevalence in hemodialysis patients: Supported. Patients reported significantly higher dialysis-specific stress than healthy controls. Stress was greater in patients on dialysis for less than 10 years. This pattern may reflect psychological adaptation over time; however, given the cross-sectional design, alternative explanations—such as the possibility that long-term patients represent a resilient “survivor” subgroup—cannot be ruled out (Chang & Kim, 2025; Sprangers & Schwartz, 1999).

H2—Anxiety and depression in hemodialysis patients: Supported. Hemodialysis patients showed higher anxiety and depression than controls, consistent with prior studies estimating that 30–40% of dialysis patients experience clinically relevant affective symptoms (Cukor et al., 2007; Feroze et al., 2012; Pereira et al., 2017). While duration of dialysis did not significantly influence these symptoms, claims about early stabilization of emotional distress should be interpreted cautiously, as longitudinal data would be needed to confirm true temporal trends.

H3—Elevated personality traits (neuroticism, psychopathic tendencies) among hemodialysis patients: Partially supported. Patients exhibited higher neuroticism and psychopathic tendencies, while paranoid traits were elevated but non-significant. These traits indicate vulnerability to emotional instability and maladaptive coping (Feroze et al., 2012; Griva et al., 2013; Nunnally & Bernstein, 1994).

H4—Relationship between dialysis duration and stress: Supported. Patients on dialysis ≥10 years reported lower stress. This may indicate adaptive mechanisms over time, but alternative explanations (e.g., survivor effects) should be considered. No significant differences in affective symptoms or personality traits were observed between dialysis duration subgroups at the time of assessment (Saracho-Rotaeche, 2013; Sprangers & Schwartz, 1999).

These findings provide a framework for understanding the interplay between situational stressors, affective symptoms, and stable personality traits in chronic dialysis patients.

### 4.2. Dialysis-Specific Stress (H1 and H4)

Consistent with H1, dialysis-specific stress was significantly higher in patients compared to controls. Stress arises from procedural demands, lifestyle restrictions, and dependency on medical staff (Palmer et al., 2013; Weisbord et al., 2005). Although patients on dialysis for <10 years reported higher stress than those treated longer, this should be interpreted tentatively. The lower stress in long-term patients could reflect adaptation or alternatively, a selection effect whereby patients who cope poorly may discontinue dialysis or experience higher morbidity/mortality.

This highlights that stress is situational and sensitive to adaptation, whereas affective symptoms may persist regardless of dialysis duration. These findings underscore the importance of early intervention to mitigate psychological burden during the initial years of treatment.

### 4.3. Anxiety and Depression (H2)

Consistent with H2, hemodialysis patients exhibited higher anxiety and depression than controls (Cukor et al., 2007; Feroze et al., 2012; Pereira et al., 2017). Approximately one-third of patients reported moderate to severe symptoms, whereas the majority of controls scored within normal ranges.

Although duration of dialysis did not significantly affect affective symptoms, any suggestion of “stabilization” over time is tentative. Longitudinal research is needed to confirm whether emotional distress truly decreases with prolonged treatment. These results reinforce the need for routine mental health screening in nephrology care, particularly during the early stages of dialysis (Chang & Kim, 2025; Saracho-Rotaeche, 2013).

### 4.4. Personality Traits (H3)

H3 examined personality differences between patients and controls. Hemodialysis patients displayed elevated neuroticism and psychopathic tendencies, indicating higher vulnerability to emotional reactivity, frustration, and impulsivity (Feroze et al., 2012; Griva et al., 2013; Kimmel, 2001). Paranoid traits showed a non-significant trend toward elevation, suggesting possible subclinical vulnerabilities.

Importantly, personality traits were relatively stable across dialysis duration, suggesting that trait-level characteristics are less influenced by chronic stress than situational factors like stress or affective symptoms (Crocker & Algina, 2006; Ebel & Frisbie, 1991; Kelley, 1939; Nunnally & Bernstein, 1994). These findings highlight the relevance of assessing individual personality profiles to guide personalized psychological interventions for patients at higher risk of maladaptive coping.

### 4.5. Limitations

This study has several limitations that warrant cautious interpretation of the findings.

First, the cross-sectional design precludes causal inference; observed differences between ESRD patients and healthy controls may reflect bidirectional influences between dialysis-related stressors, affective symptoms, and enduring personality-related tendencies rather than direct causal effects.

Second, the sample size was modest (n = 30 per group), which limits statistical power, reduces the stability of effect size estimates, and constrains generalizability to broader and more heterogeneous ESRD populations. Consistent with this limitation, some effect size estimates were associated with relatively wide confidence intervals, indicating limited precision and highlighting the need for replication in larger samples.

Third, multiple independent statistical comparisons were conducted across several psychological outcomes. No formal correction for multiple testing was applied, given the exploratory nature of the study and the risk of increasing Type II error in small samples. However, this approach may increase the risk of Type I error, and therefore the findings should be interpreted with appropriate caution.

Fourth, the reliance on self-report measures (HADS, PNP, Stress Questionnaire) introduces the potential for response biases, including social desirability and recall bias (Crocker & Algina, 2006; Nunnally & Bernstein, 1994).

Fifth, potentially important confounding variables—including prior psychiatric history, psychotropic medication use, comorbid medical conditions, socioeconomic status, and social support—were not controlled for and may have influenced psychological outcomes.

Sixth, the Dialysis-Specific Stress Questionnaire, although demonstrating excellent internal consistency and preliminary psychometric validation, was validated in a relatively small pilot sample (n = 30). While item analysis and reliability indicators supported its internal consistency and discrimination capacity, further validation in larger and more diverse samples is necessary to confirm its construct validity, factor structure, and generalizability. Therefore, findings related to dialysis-specific stress should be interpreted with appropriate caution.

Seventh, sampling bias may limit generalizability. Healthy controls were recruited from university and corporate settings, which may not be socioeconomically comparable to the clinical group. This could influence observed group differences.

Eighth, online data collection via Google Forms introduces additional considerations, particularly in a medical population. Factors such as supervision, digital literacy, environmental distractions, and variability in attention during questionnaire completion may have affected responses and should be acknowledged.

Finally, although validated instruments were used and the inclusion of a Romanian sample represents a strength, cultural, healthcare system, and contextual factors may limit the direct generalizability of these findings to other populations.

Despite these limitations, the study provides preliminary evidence supporting a multidimensional model of psychological functioning in ESRD, illustrating how dialysis-related stress, emotional distress, and personality-related vulnerability traits may co-occur and interact in patients undergoing chronic hemodialysis. These findings should be considered exploratory and hypothesis-generating, and they underscore the need for larger, longitudinal studies to clarify causal pathways and clinical implications.

### 4.6. Future Directions

Future research should aim to expand and deepen the understanding of psychological functioning in ESRD patients. Longitudinal designs are recommended to track the trajectories of dialysis-specific stress (H1, H4), anxiety and depression (H2), and personality adaptation (H3) over time, identifying periods of heightened vulnerability and potential adaptation (Chang & Kim, 2025; Saracho-Rotaeche, 2013). Larger, multicenter studies with diverse ESRD populations would enhance generalizability and allow for subgroup analyses by demographic and clinical characteristics.

Moreover, future studies should examine the efficacy of tailored psychological interventions, including cognitive–behavioral therapy, mindfulness-based stress reduction, and group support programs, particularly for patients demonstrating high neuroticism or maladaptive coping patterns. Integrating clinician-rated diagnostic tools with validated self-report measures would strengthen assessment validity and provide a more comprehensive profile of ESRD patients’ psychological health (Field, 2013; George & Mallery, 2003; Thurstone, 1931).

Ultimately, such research could inform personalized intervention strategies, optimizing mental health outcomes and treatment adherence in ESRD patients undergoing long-term hemodialysis.

## 5. Conclusions

This study provides evidence that chronic hemodialysis is associated not only with elevated emotional distress but also with a distinct psychological vulnerability profile characterized by the interaction between situational stressors and stable personality-related predispositions. This finding is consistent with previous research documenting high prevalence rates of anxiety, depression, and psychological symptom burden among patients with end-stage renal disease undergoing dialysis (Feroze et al., 2012; Palmer et al., 2013; Weisbord et al., 2005).

Importantly, the results suggest that dialysis-specific stress represents a dynamic and potentially modifiable psychological burden, particularly during the earlier stages of treatment, whereas emotional symptoms and personality-related tendencies may reflect more stable psychological patterns. This observation aligns with previous findings showing that psychosocial adaptation varies according to dialysis duration and is influenced by patients’ psychological adjustment processes (Chang & Kim, 2025; Kimmel, 2001).

From a clinical perspective, these findings support the importance of integrating routine psychological assessment into nephrology care. Beyond identifying anxiety and depression, assessing personality-related vulnerability factors may provide additional insight into individual differences in emotional adjustment and coping. Previous studies have shown that personality traits and coping mechanisms significantly influence psychological well-being, treatment adherence, and quality of life in dialysis patients (Saracho-Rotaeche, 2013; Soponaru et al., 2016; Touil et al., 2023).

Furthermore, the present findings contribute to the understanding of psychological adaptation in chronic illness by supporting a multidimensional framework in which emotional distress reflects both situational stress exposure and enduring psychological characteristics. This perspective is consistent with the broader psychosocial model of end-stage renal disease, which emphasizes the role of psychological and behavioral factors in patient adjustment and outcomes (Cukor et al., 2007; Kimmel, 2001; Lu et al., 2024).

In terms of generalization, although the findings should be interpreted with caution due to the sample size and single-center design, the observed relationships between dialysis-related stress, emotional distress, and personality vulnerability are consistent with previous research in hemodialysis populations (Ebel & Frisbie, 1991; Soponaru et al., 2016; Weisbord et al., 2005).

Finally, these results highlight the clinical value of early identification of psychological vulnerability and support the implementation of targeted psychosocial and psychoeducational interventions. Such interventions have been shown to reduce psychological distress and improve quality of life in patients undergoing maintenance hemodialysis (Zigmond & Snaith, 1983). Integrating psychological screening and intervention into routine nephrology care may therefore improve emotional adjustment and overall patient outcomes.

## Figures and Tables

**Table 1:** Comparison of anxiety and depression scores between hemodialysis patients and controls.

Outcome	Group	Mean	SD	t(df)	*p*	Cohen’s *d*	95% CI
Anxiety	Hemodialysis	10.93	4.77	2.86 (58)	0.006	0.70	0.18–1.22
	Control	8.20	2.81				
Depression	Hemodialysis	10.90	5.01	4.57 (58)	<0.001	1.26	0.71–1.81
	Control	5.67	3.04				

**Table 2:** Comparison of psychological traits between hemodialysis patients and control group.

Trait	Group	Mean	SD	t(df)	*p*	Cohen’s *d*	95% CI	% T > 70
Neuroticism	Hemodialysis	65.77	11.45	2.29 (58)	0.025	0.58	0.06–1.10	57% (n = 17)
	Control	59.13	11.37					23% (n = 7)
Paranoid traits	Hemodialysis	56.40	18.12	1.29 (58)	0.202	0.34	−0.17–0.85	40% (n = 12)
	Control	51.30	11.55					17% (n = 5)
Psychopathy-related traits	Hemodialysis	62.57	8.73	2.76 (58)	0.008	0.82	0.29–1.35	37% (n = 11)
	Control	53.97	12.04					13% (n = 4)

**Table 3:** Statistical comparison of stress scores between hemodialysis patients and controls.

Group	Mean	SD	N	t(df)	*p*	Cohen’s *d*	95% CI
Hemodialysis	62.40	20.83	30	5.84 (58)	<0.001	1.51	0.93–2.09
Control	37.23	11.14	30				

**Figure A1:**
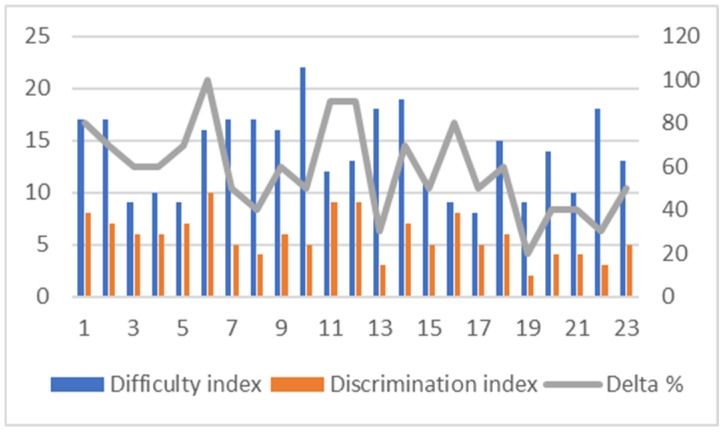
Item analysis for stress questionnaire validation. Item analysis based on difficulty index, discrimination index, and delta percentage indicates that the questionnaire demonstrates an overall good quality, effectively differentiating respondents’ performance, with a few items requiring revision to enhance validity in a healthcare assessment context.

## Data Availability

Data is contained within the article. The raw data supporting the conclusions of this article can be made available by the authors upon request.

## Abbreviations

The following abbreviations are used in this manuscript:

ESRD	End-stage renal disease
HRQoL	Health-Related Quality of Life
H1	Specific hypothesis 1
H2	Specific hypothesis 2
H3	Specific hypothesis 3
H4	Specific hypothesis 4

## Appendix A. Instruments Used in the Study

The Hospital Anxiety and Depression Scale (HADS)
  Pichot Neuroticism and Psychopathy Questionnaire (PNP)
  Dialysis-Specific Stress Questionnaire

1. The Hospital Anxiety and Depression Scale (HADS)

Introductory section

We kindly invite you to take a few moments to complete the following questionnaire, developed as part of a research study conducted at the National University of Science and Technology POLITEHNICA Bucharest.

The purpose of the instrument is to assess the emotional well-being of individuals by identifying symptoms of anxiety and depression. Emotional states can have a significant impact on physical health, and understanding these feelings can enable healthcare providers to offer better, more comprehensive care. This questionnaire is designed to help your medical team better understand how you are feeling emotionally.

Participation in this study is entirely voluntary. By continuing with the questionnaire, you acknowledge that you understand the nature and goals of the study and agree to take part. You may choose to withdraw at any time prior to submitting your responses, without providing any explanation and without experiencing any negative consequences.

The results of this study will be used only for academic and scientific purposes. No personal data, such as names, email addresses, IP addresses, or other identifying information, will be collected. Your responses will remain completely anonymous and confidential.

All data will be analysed in aggregate form at the group level. Individual responses will not be published or interpreted separately in any scientific communication. Access to the collected data will be limited to members of the research team.

All information will be securely stored online via the Google Drive platform, which has implemented the necessary measures to comply with Regulation (EU, 2016) 2016/679 on the protection of natural persons regarding the processing of personal data and the free movement of such data (General Data Protection Regulation—GDPR).

We appreciate your time and contribution to this research!

Instructions: Please read each statement carefully and select one response that best describes how you have felt during the past week. Do not spend too much time thinking about each item, your immediate reaction is likely to be more accurate than a carefully considered response.

Items:

1. I feel tense and wound up.
  - Most of the time (3)
  - Often (2)
  - From time to time (1)
  - Not at all (0)
2. I feel slowed down in my daily activities.
  - All the time (3)
  - Very often (2)
  - Sometimes (1)
  - Not at all (0)
3. I still enjoy things as much as I used to.
  - Just as much (or even more) (0)
  - I enjoy things only occasionally (1)
  - Not quite as much (2)
  - Hardly at all or not at all (3)
4. I get a sort of frightened feeling as if something bad is about to happen in my stomach.
  - Not at all (0)
  - Occasionally (1)
  - Most of the time (2)
  - Very often (3)
5. I get sudden feelings that something terrible is about to happen.
  - Yes, very strongly (3)
  - Yes, but not too strongly (2)
  - I get a vague feeling, but I’m not really worried (1)
  - Not at all (0)
6. I have lost interest in my appearance.
  - Definitely (3)
  - Quite often (2)
  - Sometimes (1)
  - Not at all (0)
7. I can laugh and see the funny side of things.
  - Just as much as always (0)
  - Not quite as much as before (1)
  - Definitely less than I used to (2)
  - Not at all (3)
8. I feel restless, as if I have to be constantly moving.
  - Yes, very much so (3)
  - To some extent (2)
  - Not very often (1)
  - Not at all, I feel calm (0)
9. Worrying thoughts keep going through my mind.
  - Most of the time (3)
  - Quite often (2)
  - From time to time (1)
  - Very rarely or never (0)
10. I look forward to the future with confidence.
  - Yes, with a lot of confidence (0)
  - Somewhat less than before (1)
  - Much less than before (2)
  - Hardly or not at all (3)
11. I feel cheerful and happy.
  - Not at all (3)
  - Rarely (2)
  - Fairly often (1)
  - Most of the time (0)
12. I get sudden feelings of panic.
  - Very often (3)
  - Quite often (2)
  - Not very often (1)
  - Almost never (0)
13. I can sit at ease and feel relaxed.
  - Yes, definitely (0)
  - Usually (1)
  - Not often (2)
  - Hardly at all (3)
14. I can enjoy reading a book or watching a TV or radio program.
  - Often (0)
  - Sometimes (1)
  - Occasionally (2)
  - Very rarely (3)

Scoring and interpretation:

The assessment is made by summing the scores for each item. Each question is rated according to the following scoring system:

**Table A1:** Item scoring method.

Item	Score 1	Score 2	Score 3	Score 4
1.	3	2	1	0
2.	3	2	1	0
3.	0	1	2	3
4.	0	1	2	3
5.	3	2	1	0
6.	3	2	1	0
7.	0	1	2	3
8.	3	2	1	0
9.	3	2	1	0
10.	0	1	2	3
11.	3	2	1	0
12.	3	2	1	0
13.	0	1	2	3
14.	0	1	2	3

Anxiety Subscale:

Items: 1, 4, 5, 8, 9, 12, 13

Total possible score: 0–21

Interpretation:

0–10 points: Normal (no clinically significant anxiety symptoms)

11–14 points: Mild anxiety

15–17 points: Moderate anxiety

18–21 points: Severe anxiety

Depression Subscale:

Items: 2, 3, 6, 7, 10, 11, 14

Total possible score: 0–21

Interpretation:

0–10 points: Normal (no signs of depression)

11–14 points: Mild depression

15–17 points: Moderate depression

18–21 points: Severe depression

**Table A2:** Scoring and Interpretation of HADS Subscales

Subscale	Items	Total Possible Score	Interpretation
Anxiety	1, 4, 5, 8, 9, 12, 13	0–21	0–10: Normal (no clinically significant anxiety symptoms); 11–14: Mild anxiety; 15–17: Moderate anxiety; 18–21: Severe anxiety
Depression	2, 3, 6, 7, 10, 11, 14	0–21	0–10: Normal (no signs of depression); 11–14: Mild depression; 15–17: Moderate depression; 18–21: Severe depression

2. Pichot Neuroticism and Psychopathy Questionnaire (PNP)

Introductory section

We kindly invite you to take a few minutes to complete the following questionnaire, administered as part of a research study conducted at the National University of Science and Technology POLITEHNICA Bucharest.

This questionnaire is designed to assess the frequency and intensity of various psychological and somatic experiences that may reflect underlying emotional or mental distress. The aim is to better understand emotional functioning, vulnerability to psychological symptoms, and the presence of potential psychopathological markers within specific populations.

Participation is entirely voluntary. By continuing with the questionnaire, you confirm that you understand the purpose and procedures of the study and agree to participate. You may discontinue participation at any point before submitting your answers, without explanation and with no negative consequences.

No identifying information such as names, email addresses, or IP addresses will be collected. All data will be treated with the highest level of confidentiality and anonymity, and results will be analysed in aggregate form only. Individual responses will not be reported or interpreted separately in any scientific publication.

Access to data will be strictly limited to members of the research team. The information collected will be stored securely on Google Drive, which complies with Regulation (EU, 2016) 2016/679 (General Data Protection Regulation—GDPR).

Your participation is greatly appreciated and contributes to the advancement of psychological research.

Thank you for your time and valuable input!

Part I. Food taste

Instructions: Please select the foods listed below that you do not like:

1. Radishes
2. Red beets
3. Celery salad or celery with mayonnaise
4. Sardines in oil
5. Canned tuna or marinated canned crab
6. Cabbage soup
7. Sorrel soup
8. Ham
9. Grilled beefsteak or tenderloin
10. Liver pâté
11. Poached eggs or sunny-side-up eggs
12. Cabbage
13. Mushrooms
14. Carrots
15. Mashed potatoes
16. Yogurt
17. Cherries
18. Peaches
19. Strawberries
20. Stewed prunes

Part II. The PNP questionnaire

Instructions:

Please read each item carefully and choose the answer that best reflects your own experience.

For each statement, indicate whether it is True or False based on how you feel or think in general. Please do not answer with a question mark (?). Use this option only if you genuinely cannot decide between True or False.

There are no right or wrong answers—your responses should reflect your personal thoughts, feelings, or experiences as accurately as possible.

**Table A3:** The PNP Questionaire

	Item	True	False
1	I sometimes suffer from violent headaches.	☐	☐
2	I sometimes laugh at crude jokes.	☐	☐
3	My daily life is full of interesting things.	☐	☐
4	There are days when everything seems to go wrong.	☐	☐
5	I often do things I later regret.	☐	☐
6	I have sometimes been left out or mistreated out of malice.	☐	☐
7	I sometimes get angry.	☐	☐
8	Sometimes, I vote for candidates I barely know.	☐	☐
9	Among my acquaintances, there are people I dislike.	☐	☐
10	A new experience almost always pulls me out of boredom.	☐	☐
11	I find it hard to make new friends.	☐	☐
12	I have feelings of inferiority that bother me.	☐	☐
13	I find it difficult to concentrate on one task or activity.	☐	☐
14	I enjoy meeting famous people because it makes me feel important too.	☐	☐
15	Sometimes my hearing is so sensitive it irritates me.	☐	☐
16	I have enemies who truly wish to harm me.	☐	☐
17	I am bothered by what others think about me.	☐	☐
18	I sometimes feel dizzy.	☐	☐
19	My parents often criticized my friendships.	☐	☐
20	In the presence of superiors, I often worry about the impression I make.	☐	☐
21	My parents and family find more flaws in me than I actually have.	☐	☐
22	I have had a nervous breakdown.	☐	☐
23	I am irritable (I get annoyed easily).	☐	☐
24	Sometimes I get lost in thought and forget where I am.	☐	☐
25	I have had more bad luck than others.	☐	☐
26	Someone holds a grudge against me.	☐	☐
27	I often find myself in upsetting situations I did not cause.	☐	☐
28	Sometimes I have crying or laughing fits that I cannot control.	☐	☐
29	Sometimes I feel happy, other times depressed, for no apparent reason.	☐	☐
30	When crowded in a tram or bus, I’m tempted not to punch a ticket.	☐	☐
31	There are days when I don’t read the newspaper.	☐	☐
32	Sometimes I lose my breath or pant without much effort.	☐	☐
33	I get nervous in elevators, trains, or tunnels.	☐	☐
34	Almost always, I feel happy.	☐	☐
35	Sometimes I’m nervous.	☐	☐
36	Sometimes I can’t fall asleep because of my thoughts.	☐	☐
37	I would be disgusted with justice if a criminal were released just due to a lawyer’s clever argument.	☐	☐
38	I feel I live less intensely than others.	☐	☐
39	No one understands me.	☐	☐
40	I am often worried about my health.	☐	☐
41	I wish I were as happy as others seem to be.	☐	☐
42	I know who is responsible for my troubles.	☐	☐
43	I have lost consciousness due to an accident or injury.	☐	☐
44	Sometimes I feel like swearing.	☐	☐
45	I often feel annoyed.	☐	☐
46	I often have bouts of diarrhea.	☐	☐
47	I experience tremors or chills.	☐	☐
48	I often daydream.	☐	☐
49	I have nightmares.	☐	☐
50	I had to stop working for a long period due to illness.	☐	☐
51	There were times I really wanted to leave my family.	☐	☐
52	I get bothered when I stutter.	☐	☐
53	Even when I’m with others, I feel lonely.	☐	☐
54	I enjoy occasional small parties.	☐	☐
55	I have strong heart palpitations.	☐	☐
56	My table manners at home are worse than when I eat with strangers.	☐	☐
57	I prefer winning at a game to losing.	☐	☐
58	Sometimes I postpone things I should do today.	☐	☐
59	If people hadn’t been hostile to me, I would have had more success.	☐	☐
60	When things go wrong, I get more blame than I deserve.	☐	☐
61	People pay more attention to my business than they should.	☐	☐
62	I rarely argue with my family members.	☐	☐
63	Some people have taken credit for my work without remorse.	☐	☐
64	I suffer from insomnia.	☐	☐
65	From time to time, I feel full of energy without any special reason.	☐	☐
66	Sometimes, when I don’t feel well, I’m in a bad mood.	☐	☐
67	I deserve more than I have now.	☐	☐
68	Sometimes I feel a pain in my heart.	☐	☐
69	At school, I was sometimes sent to the principal for misbehavior.	☐	☐
70	Some people have made me feel fear that later proved unjustified.	☐	☐
71	I’m sure I’ll never be lucky.	☐	☐
72	My family disagrees with the career I’ve chosen.	☐	☐
73	Almost all my relatives like me.	☐	☐
74	I often have aches and pains.	☐	☐
75	My feelings are easily hurt.	☐	☐
76	I’m against giving alms to beggars.	☐	☐
77	Sometimes I think about things too horrible to mention.	☐	☐
78	I often feel very miserable.	☐	☐
79	Almost everyone would use dishonest means to gain advantage rather than miss an opportunity.	☐	☐
80	I’m haunted for a long time by a humiliating experience.	☐	☐
81	Sometimes I don’t tell the whole truth.	☐	☐
82	I have often been unfairly punished.	☐	☐
83	I think I have as pleasant a family life as most people I know.	☐	☐

Part III. Verbal Associations

Instructions:

Below you will find a list of words you are familiar with. In the first column, you will see a word written in uppercase letters, followed by two lowercase words in the next two columns.

Take, for example, the word SCORCHED. Now look at the two associated words: burnt and poor.

The word scorched might make you think more of burnt or perhaps of poor. Please select the word that you feel is more closely associated in your mind with the capitalized word.

There are no right or wrong answers, because each association is equally acceptable.

You simply need to look at the two lowercase words and select the one that, for you, has the strongest mental connection to the uppercase word.

Work quickly, and don’t spend too much time reflecting on any one word.

Please make sure you do not skip any lines.

**Table A4:** Verbal Associations

Main Word (UPPERCASE)	Option A	Option B
DIGNIFIED	Snob	thoughtful
SLEEP	nightmare	bed
CHILD	abandoned	small
HUNGRY	thirsty	heart
FAST	hurricane	slow
FOOD	stomach	poison
GATHERING	crowd	self
BITTER	medicine	sweet
TOOTH	Pain	mechanism
BED	blanket	ill
HEAD	migraine	face

Scoring:

Test 1—Food Preferences:

Count the number of selected food items. This score is recorded in the “Food Taste” section on the profile sheet.

Test 2—Questionnaire:

Apply the four scoring grids corresponding to:

- Neuroticism (N)
- Lie Scale (L)
- Paranoia (Pa)
- Psychopathy (Ps)

Enter the resulting scores in the appropriate boxes on the profile sheet.

Test 3—Verbal Associations (Similar Words):

Count the number of incorrect associations (wrong choices), and assign one point for each. This score is entered in the Verbal Association section on the profile sheet.

Interpretation Guidelines

Use the profile corresponding to the participant’s educational level.

Add the T scores obtained for:

Food Taste

Verbal Association

2 × Nonverbal Probability

Record the sum in the profile sheet. This composite score represents the subject’s neurotic tendency.

The standardized scores (T scores) recorded for Pa and Ps in the profile sheet represent the corresponding tendencies.

Connect the points on the profile chart representing the three tendencies.

Subjects scoring above T = 70 are considered to show pathological tendencies.

If a subject scores above T = 70 only on the Lie Scale, they may be suspected of paranoia.

**Table A5:** Raw Scores and Corresponding Stanines for PNP Subscales.

Raw Scores			Stanines
N	Pa	Ps	
17–30	12–20	14–20	1
12–16	9–11	12–13	2
8–11	7–6	10–11	3
5–7	5–6	7–9	4
3–4	3–4	6	5
2	2	5	6
1	1	4	7
0	0	3	8
0	0	0–2	9

Scoring Grids

I. Questionnaire Scales

Lie Scale (L)—Answered as False: Items: 2, 7, 8, 9, 14, 29, 30, 44, 54, 56, 57, 58, 66, 77, 81.

Neuroticism Scale (N)—Answered as True: Items: 1, 11, 12, 18, 21, 22, 23, 24, 29, 32, 33, 35, 36, 38, 40, 43, 45, 46, 49, 50, 52, 55, 64, 68, 74, 75, 78, 80.

Paranoia Scale (Pa)—Answered as True: Items: 4, 6, 15, 16, 25, 26, 27, 28, 39, 53, 59, 60, 61, 63, 67, 70, 71, 72, 82. Answered as False: Item 10.

Psychopathy Scale (Ps)—Answered as True: Items: 5, 13, 17, 19, 20, 39, 41, 42, 51, 59, 69, 72. Answered as False: Items 3, 34, 37, 62, 65, 73, 76, 83.

II. Food Preferences Test

Assign 1 point for each food item marked.

III. Verbal Associations

Assign 1 point for each correct association according to the key below:

SCISSORS—nurse

POOR—individual

FRIEND—hypocrite

DIGNIFIED—snob

SLEEP—nightmare

CHILD—found

HEAD—headache

HANDS—sweaty

STOMACH—pain

SLOW—fast

SATISFIED—unsatisfied

UNHAPPY—yes

HEAVY—heart

HUNGRY—heart

FOOD—poison

FAST—slow

GATHERING—self

BITTER—sweet

TOOTH—pain

BED—sick

3. Dialysis-Specific Stress Questionnaire

Introductory Section

We kindly ask you to take a few minutes to complete the following Dialysis-Specific Stress Questionnaire, conducted as part of a research study at the National University of Science and Technology POLITEHNICA Bucharest.

The purpose of this questionnaire is to evaluate your level of stress related to various aspects of your daily life and medical treatment. It aims to identify stress factors that may affect your well-being, concentration, emotions, and overall mental health.

Participation in this study is entirely voluntary. By proceeding with the questionnaire, you indicate that you understand the nature and purpose of the study and consent to participate. You may withdraw at any time before submitting your responses, without any need to provide justification and without any negative consequences.

The results of this study will be used exclusively for academic and scientific purposes. No names, email addresses, IP addresses, or any other personal identifiers will be collected. All responses will remain completely anonymous and confidential.

All data will be analysed at the group level, and no individual responses will be presented or interpreted separately in any scientific publication. Only members of the research team will have access to the recorded information.

Throughout the study, data will be securely collected and stored online via the Google Drive platform, which complies with Regulation (EU, 2016) 2016/679 on the protection of natural persons with regard to the processing of personal data and the free movement of such data (General Data Protection Regulation—GDPR).

Thank you for your time and valuable contribution!

**Table A6:** Dialysis-Specific Stress Questionnaire

Item No.	Question	Always	Often	Normal	Sometimes	Very Rarely/Never
1	Do you find traveling to the hospital tiring or difficult?					
2	Do malfunctions of the dialysis machine cause problems for you?					
3	Have you experienced episodes of aggression or violence?					
4	Do you have difficulty concentrating?					
5	Do you feel isolated or lonely, even among friends?					
6	Do you frequently feel restless or fearful?					
7	Do you have difficulty performing professional or daily activities?					
8	Do you feel the need for an assistant to be present during dialysis?					
9	Do dietary and fluid restrictions bother you?					
10	Do you often feel nervous or irritated?					
11	Do you sometimes feel so discouraged that nothing could cheer you up?					
12	Are things going the way you want in your daily life?					
13	Do minor annoyances irritate you more now than before the dialysis?					
14	Do personal fears influence your mental state?					
15	Do you feel agitated, disoriented, or confused?					
16	Do you feel that nothing has value or meaning to you?					
17	Do you feel helpless?					
18	Do you have a feeling of emptiness inside?					
19	Do you believe your health condition will worsen?					
20	Do you consider dialysis itself a significant source of stress?					

Scoring Instructions:

For each item, responses are scored from 1 to 5 points, depending on the chosen answer:

Always = 5 points

Often = 4 points

Normal = 3 points

Sometimes = 2 points

Very Rarely/Never = 1 point

The total score is calculated by summing the points for all items.

**Table A7:** Scoring Instructions.

Item	Always	Often	Normal	Sometimes	Very Rarely/Never
1.	5	4	3	2	1
2.	5	4	3	2	1
3.	5	4	3	2	1
4.	5	4	3	2	1
5.	5	4	3	2	1
6.	5	4	3	2	1
7.	5	4	3	2	1
8.	5	4	3	2	1
9.	5	4	3	2	1
10.	5	4	3	2	1
11.	5	4	3	2	1
12.	1	2	3	4	5
13.	5	4	3	2	1
14.	5	4	3	2	1
15.	5	4	3	2	1
16.	5	4	3	2	1
17.	5	4	3	2	1
18.	5	4	3	2	1
19.	5	4	3	2	1
20.	5	4	3	2	1

Interpretation Scale:

**Total Score Range**	**Stress Level Interpretation**
20–40	Low stress
41–60	Moderate stress
61–80	High stress
81–100	Very high stress

## Appendix B. Psychometric Validation of the Dialysis-Specific Stress Questionnaire

The 23-item Dialysis-Specific Stress Questionnaire was developed specifically to measure the multifaceted emotional, behavioral, and physical stress experienced by chronic hemodialysis patients. Items are rated on a 5-point Likert scale (Always, Often, Normal, Sometimes, Rarely/Never), capturing three primary symptom domains:

- Mental and behavioral symptoms (e.g., diminished concentration, apathy, irritability, memory problems),
- Emotional symptoms (e.g., social withdrawal, anxiety, depressive symptoms, medication adherence),
- Physical symptoms (e.g., fatigue, sleep disturbances, gastrointestinal and cardiovascular complaints).

Additionally, the questionnaire includes stressors specific to hemodialysis, such as transportation difficulties, dialysis equipment malfunctions, activity restrictions, strict dietary/fluid guidelines, and the presence of medical personnel during treatment.

Preliminary psychometric validation was conducted on a pilot sample of 30 chronic hemodialysis patients. Participants were stratified into three equal groups based on total scores (high, medium, low scorers) to assess item difficulty and discrimination. The Delta coefficient (Δ) was calculated for each item to evaluate its sensitivity in distinguishing between high- and low-stress individuals, using established formulas adapted from psychometric theory.

The questionnaire’s psychometric properties were evaluated following classical test theory (CTT) principles in a pilot sample of 30 chronic hemodialysis patients (Anastasi & Urbina, 1997; Nunnally & Bernstein, 1994). The validation steps included:

Item difficulty indices were calculated to identify items that were excessively easy or difficult, with such items being considered for removal based on established guidelines (Ebel & Frisbie, 1991).

1. Item discrimination indices, which measure the ability to distinguish between respondents with high and low overall scores, were assessed following established recommendations, resulting in the removal of items with poor discrimination (Crocker & Algina, 2006; Kelley, 1939).
2. Distractor analysis was performed on multiple-choice items to remove ineffective distractors that failed to discriminate between respondents.
3. Item-total correlations (Pearson’s r) were computed to evaluate each item’s consistency with the overall scale; items with correlations below 0.20 were discarded as seen in Figure A1 (Field, 2013).

Based on these criteria, 3 items were removed, resulting in a final 20-item scale. Internal consistency was excellent, with Cronbach’s alpha = 0.938, indicating high reliability (George & Mallery, 2003). The pilot data used for validation purposes were not included in the main statistical analyses comparing the clinical and control groups.

This structured validation process ensures the content validity, internal consistency, and discrimination ability of the instrument, making it suitable for measuring stress levels in patients undergoing chronic hemodialysis.

It is worth noting that the validation was conducted on a relatively small pilot sample (n = 30), which may limit the generalizability of psychometric properties. Future studies with larger samples would be beneficial to confirm these findings.
